# Olanzapine-induced lipid disturbances: A potential mechanism through the gut microbiota-brain axis

**DOI:** 10.3389/fphar.2022.897926

**Published:** 2022-08-05

**Authors:** Zhenyu Zhu, Yuxiu Gu, Cuirong Zeng, Man Yang, Hao Yu, Hui Chen, Bikui Zhang, Hualin Cai

**Affiliations:** ^1^ Department of Pharmacy, The Second Xiangya Hospital, Central South University, Changsha, China; ^2^ Institute of Clinical Pharmacy, Central South University, Changsha, China; ^3^ International Research Center for Precision Medicine, Transformative Technology and Software Services, Hunan, China; ^4^ School of Pharmacy, Changsha Medical University, Changsha, China; ^5^ School of Pharmacy, Hunan University of Medicine, Changsha, China

**Keywords:** gut microbiota-brain axis, gut microbiome, vagus nerve, lipid disturbances, olanzapine

## Abstract

**Objective:** Long-term use of olanzapine can induce various side effects such as lipid metabolic disorders, but the mechanism remains to be elucidated. The gut microbiota-brain axis plays an important role in lipid metabolism, and may be related to the metabolic side effects of olanzapine. Therefore, we explored the mechanism by which olanzapine-induced lipid disturbances through the gut microbiota-brain axis.

**Methods:** Sprague Dawley rats were randomly divided into two groups, which underwent subphrenic vagotomy and sham surgery. Then the two groups were further randomly divided into two subgroups, one was administered olanzapine (10 mg/kg/day) by intragastric administration, and the other was administered normal saline by intragastric administration (4 ml/kg/day) for 2 weeks. The final changes in lipid parameters, gut microbes and their metabolites, and orexin-related neuropeptides in the hypothalamus were investigated among the different groups.

**Results:** Olanzapine induced lipid disturbances as indicated by increased weight gain, elevated ratio of white adipose tissue to brown adipose tissue, as well as increased triglyceride and total cholesterol. Olanzapine also increased the Firmicutes/Bacteroides (F/B) ratio in the gut, which was even aggravated by subphrenic vagotomy. In addition, olanzapine reduced the abundance of short-chain fatty acids (SCFAs) metabolism related microbiome and 5-hydroxytryptamine (5-HT) levels in the rat cecum, and increased the gene and protein expression of the appetite-related neuropeptide Y/agouti-related peptide (NPY/AgRP) in the hypothalamus.

**Conclusion:** The abnormal lipid metabolism caused by olanzapine may be closely related to the vagus nerve-mediated gut microbiota-brain axis.

## 1 Introduction

Schizophrenia is a complex and severe mental illness, that affects nearly 1% of the world’s population, and it is one of the top ten causes of disability in the world (Marder and Cannon, 2019). Currently, atypical antipsychotic drugs (AAPDs) are the mainstay of treatment for schizophrenia, and the metabolic side effects have become a key factor in reduced quality of life and increased relapse of patients (Grajales et al., 2019). Olanzapine (OLZ), one of the representative AAPDs, is prone to causing weight gain, obesity, hyperglycemia and dyslipidemia after long-term use, and these side effects occur more frequently in children and women (Visconti et al., 2019). The metabolic side effects also greatly increase the risks in comorbidities of type two diabetes and cardiovascular disease (Anyanwagu et al., 2018). Administration of OLZ can promote food intake, alter energy expenditure and metabolic levels, and induce insulin resistance, ultimately having negative impact on the parameters of body weight, fasting blood glucose and triglyceride levels (Teff and Kim, 2011; Lord et al., 2017; Maruvada et al., 2017; Ballon et al., 2018; Bush et al., 2018; Skonieczna-Zydecka et al., 2019). Notably, overweight patients with schizophrenia are twice as likely to discontinue antipsychotic drugs as patients with normal weight, and discontinuation is a common cause of symptoms recurrence (Rummel-Kluge et al., 2008). Therefore, how to investigate the underlying mechanisms of AAPD-induced metabolic side effects and to form corresponding coping strategy are urgently needed.

The exact mechanisms by which AAPDs cause weight gain and lipid disturbances are complex. According to previous findings, AAPDs can accelerate peripheral adipogenesis by regulation of sterol-regulatory element binding protein (SREBP) (Ader et al., 2005). Another recent study further indicates that insulin-induced gene (INSIG), and progesterone receptor membrane component 1 (PGRMC1) as the upstream regulatory factors of SREBP, can be inhibited by AAPDs and consequently produce disturbances in lipid metabolism by affecting the PGRMC1/INSIG/SREBP pathway in the liver (Cai et al., 2015). Specifically, available evidence shows that altered levels of adenosine 5′-monophosphate-activated protein kinase (AMPK) and gastrointestinal peptide (such as glucagon-like peptide-1, GLP-1) can contribute to the lipid metabolic side effects induced by OLZ (Ikegami et al., 2013; Teff et al., 2013; Li et al., 2016). It has been suggested that OLZ significantly increased blood lipid levels and hepatic lipid accumulation by increasing the expression of mammalian target of rapamycin complex-1 (mTORC1) and p-mTORC1 (Liu et al., 2019). In addition, OLZ can increase body fat percentage in rats through oxidative stress signaling, which is reversable by antioxidants (Bilgic et al., 2017; Isaacson et al., 2020).

The metabolic disorders are also closely related to the central effects produced by OLZ (Yang et al., 2007). Current studies have shown that the possible mechanism is related to the increased antagonism of 5-HT_2C_ and H_1_ receptors in the hypothalamus (Casey and Zorn, 2015). Although OLZ exerts its therapeutic effects on various neurotransmitter systems (Huang et al., 2014), the changes in 5-HT seem to play a major role in appetite control, especially that the involvement of endogenous hypothalamic 5-HT can cause satiety during or after meals (Halford et al., 2005). Meanwhile, 5-HT suppresses appetite and promotes energy expenditure mainly by stimulating the sympathetic drive of brown adipose tissue (Tecott, 2007; Yabut et al., 2019), promotes the release of insulinotropic signals in white adipose tissue to reduce lipolysis, and alters the process of *de novo* adipogenesis in the liver (Crane et al., 2015; Oh et al., 2015). Furthermore, it has been shown that exogenous substances, such as glucose, fatty acids, and drugs, can alter intestinal 5-HT release in the duodenum by affecting the function of microbiota (Walther et al., 2003). In recent years, the gut microbiota has been found to be closely related to metabolic status (Zmora et al., 2019). Long-term administration of OLZ induces weight gain and lipid deposition in rats, whereas coadministration with antibiotics shows the opposite (Davey et al., 2013). In support, OLZ administration can increase the proportion of obesity-related bacteria in the and cause weight gain, and the combination with prebiotics can alleviate this side effect (Morgan et al., 2014; Kao et al., 2018; Kao et al., 2019; Abbas et al., 2022). In addition, targeting the *Akkermansia muciniphila* in intestinal microorganisms can partially correct abnormal blood glucose caused by OLZ (Huang et al., 2021). The abovementioned evidence reveals that the gut microbiota may play a pivotal role in the metabolic disorders caused by OLZ, but the specific mechanisms remain to be explored in depth.

The review by Coccurello and Moles (2010) has addressed comprehensively the clinical impact of AAPD-induced metabolic derangement and the hypotheses for related obesogenic and diabetogenic mechanisms. However, at that time, the key role of gut microbiota was largely undervalued or unknown, whereas the present attention towards gut microbiota-brain axis bidirectional communication allows the exploration of additional mechanistic hypotheses. The gut microbiota assists with the absorption of substances and the storage of energy (Eckburg et al., 2005; Duca and Lam, 2014), and can affect brain function through “The Microbiota-Gut-Brain Axis” (Mayer et al., 2015; Cryan et al., 2019). The gut microbiota and the brain can communicate with each other through signal conduction by various channels, including immunity, vagus nerve and enteric nervous system (Cryan et al., 2019). Among them, the most important one is the vagus nerve, which can innervate the entire intestine through vagus nerve afferent neurons and participate in the regulation of satiety, intestinal homeostasis and the inflammatory response (Singh et al., 2020). In addition, short-chain fatty acids (SCFAs) as the main metabolites produced by gut microbiota, have also been found to affect the colonic environment, blood sugar and blood lipid levels (Yamashita et al., 2009; Canfora et al., 2015). It is noteworthy that SCFAs have also been proven to promote the secretion of 5-HT by stimulating enterochromaffin cells, and to stimulate the vagus nerve through 5-HT_3_ receptors on the vagus nerve endings, thereby regulating the body’s lipid metabolism (Browning, 2015; Reigstad et al., 2015). The vagus nerve serves as the physiological connection between the GM and the central nervous system, which provides the basis for regulating appetite through the Microbiota-Gut-Brain Axis (Cork, 2018). After receiving serotonergic signals from the gut, the brain regulates appetite through the expression of neuropeptide Y/agouti-related peptide (NPY/AgRP) in the hypothalamus. As appetite-stimulating peptides, NPY and AgRP can induce food intake and reduce energy expenditure (Schwartz et al., 2000; Savontaus et al., 2002).

In the present study, we aimed to investigate one possible additional mechanism responsible of these important unwanted and highly deleterious metabolic side effects. It is hypothesized that gut microbiota-brain axis is involved in the metabolic effects induced by OLZ, and the changes in the gut microbiota can lead to a consequent increase in the expression of orexin-stimulating NPY/AgRP in the hypothalamus, and lipid disturbances in the periphery.

## 2 Materials and methods

### 2.1 Drugs and reagents

Olanzapine was purchased from Shanghai McLean Biochemical Technology Co., Ltd. (Shanghai, China) and dissolved in 0.9% saline at 2.5 mg/ml, adjusting to pH 6.5 with citric acid. CCK-8 was obtained from Sigma-Aldrich^®^ (Shanghai, China) and freshly prepared in PBS solution (4 μg/ml). The rat 5-HT assay kits were provided by Jiangsu KETE Biotechnology Co., LTD. (Yancheng, China). BCA protein quantitative assay kit and radioimmunoprecipitation assay buffer containing phenylmethyl sulfonyl fluoride were purchased from Boster Biological Technology Co., Ltd. (California, United States). The primary antibody against NPY was purchased from Cell Signaling Technology Co., Ltd. (Danvers, United States). The primary antibody against AgPY was purchased from Santa Cruz Biotechnology Co., Ltd. (Shanghai, China). Primary antibody against β-actin, and all secondary antibodies were purchased from Proteintech Group, Inc. (Wuhan, China).

### 2.2 Animals and treatments

**FIGURE 1:**
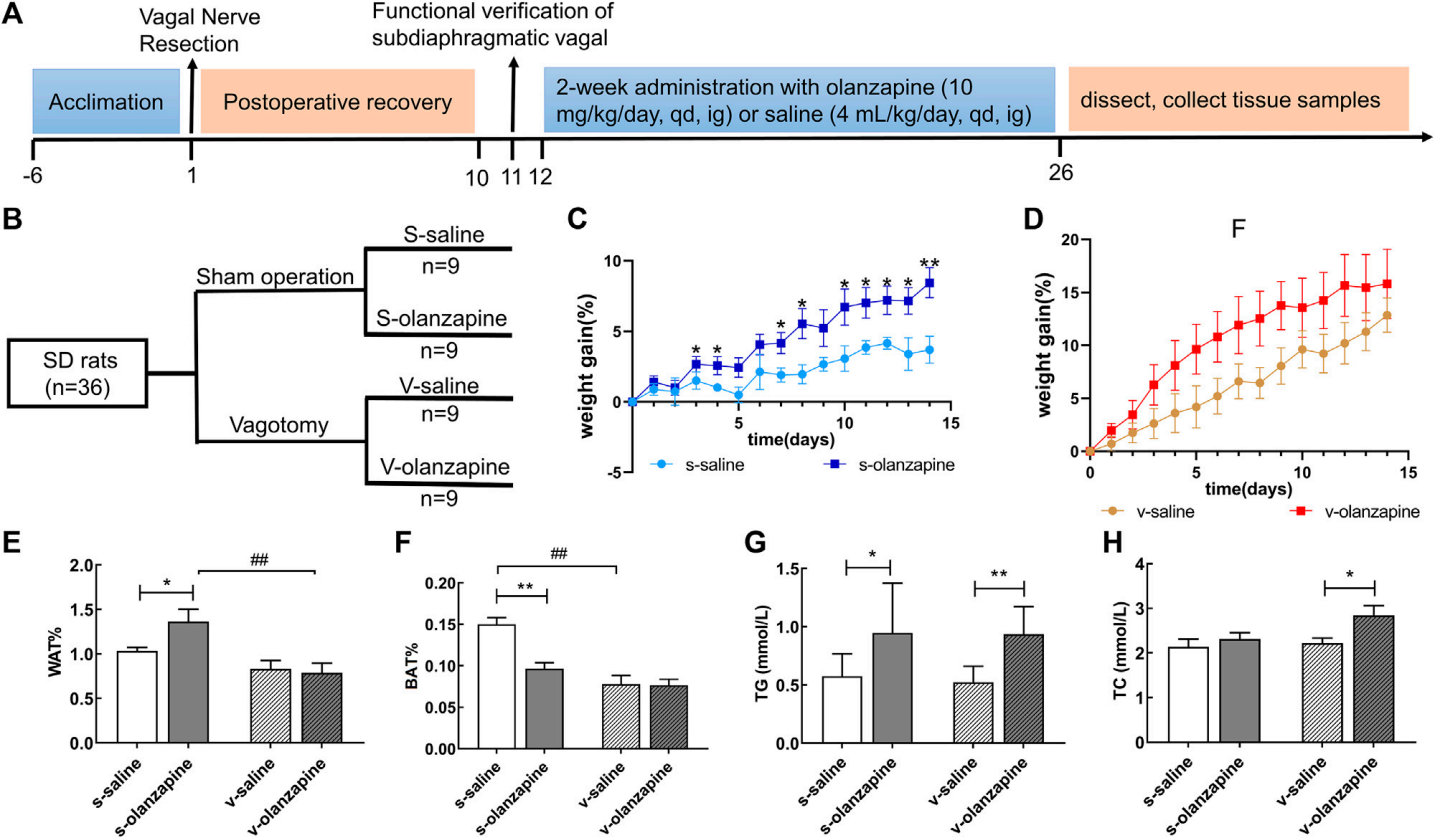
The experiment was designed following the timeline **(A)** and grouping **(B)**. The results of lipid parameters are indicated as: weight gain (%) changes over time in the sham groups **(C)** and in the vagotomy groups **(D)**; the weight percentage of the white adipose tissue in the groin **(E)** and of the brown adipose tissue between the scapula **(F)**; and the serum levels of **(G)** triglyceride (TG) and **(H)** total cholesterol (TC) in different groups. Data are represented as the mean ± SD, *p < 0.05, **p < 0.01 and ^##^ *p* < 0.01.

Adult female Sprague Dawley (SD) rats, weighing 200 ± 10 g, were provided by the Hunan STA Laboratory Animal Co., LTD. (Xinjiang China) [No. SCXK (xiang) 2019-0004]. The experimental flow is depicted in Figure 1A. The rats were free to access to commercial rat chow (SLAC Laboratory Animal, Shanghai, China) and water, and were reared under 12-h light/dark cycle environment at approximately 24–26°C and in relatively humidity of 40%–60%. All the experimental procedures conformed to the Declaration of Helsinki and were approved by the Experimental Animal Ethics Committee of the Department of Experimental Animals, Central South University (No. 2019sydw0259).

Forty female SD rats were randomly divided into a sham operation group (*n* = 22) and a vagotomy group (*n* = 18) according to body weight. After 1 week of acclimatization, rats in the sham operation group were sham-operated, and rats in the vagotomy group were performed with subphrenic vagotomy. The specific operation procedure of vagal nerve resection was as follows (Klarer et al., 2014): after being anesthetized by intraperitoneal injection of 3% sodium pentobarbital (1 ml/kg), the rat skin and abdominal wall were incised along the midline of the abdomen to expose the stomach and esophagus, in order to allow searching for the trunk of vagus nerve. The subphrenic vagus nerve trunk was resected and all vague nerve branches were transected. The wound was sutured and disinfected. The sham operation group underwent the same operation procedure except that the vagus nerve was not resected. After 10 days of postoperative recovery, to test the successfulness of vagotomy, rats were fasted overnight (12 h) and intraperitoneally injected with 4 μg/kg CCK-8 in PBS solution. After 2 h, the rats were given food and monitored for the extent of food intake (Klarer et al., 2014). Within a timeframe of 30 min after CCK-8 injection, the sham group with intact vagal nerve typically consumed 25%–40% less food than the vagotomy group (Klarer et al., 2014). Therefore, the rats in the vagotomy group which had less than 25% of food intake reduction in food intake in the first 30 min were excluded. Finally, 4 out of 22 vagotomy rat animals had to be culled. Then, each large group was randomly divided into two subgroups (each *n* = 9, Figure 1B), and the body weight of the rats was weighed every day. Each rat was administrated with olanzapine by oral gavage at 10 mg/kg/day or 0.9% saline for 2 weeks before sacrifice.

On Day 26, after fasting for 12 h, the rats were anesthetized by intraperitoneal injection of 3% sodium pentobarbital (1 ml/kg). The truncal blood was collected using vacuum blood collection tubes and centrifuged for 10 min (3,000 r/min, 4°C) to obtain serum samples. Then the white adipose tissue (WAT) in the bilateral groin, the brown adipose tissue (BAT) between the scapulae, and the contents of the cecum and fecal samples were collected from the body and quickly frozen in liquid nitrogen. Rat heads were rapidly dissected and the tissue of hypothalamus was removed on ice trays and snap frozen in liquid nitrogen. All the above-mentioned rat samples were stored in −80°C refrigerator before analysis.

### 2.3 Lipid parameters, olanzapine concentration and microbiota metabolites

The rats were weighed every day, and the percentage of body weight gain was calculated as weight gain% (WG%) = (weight on the day-initial weight)/initial weight*100%. The percentage of two kinds of adipose tissue in the body weight was calculated as WAT% and BAT%. The serum levels of triglyceride (TG) and total cholesterol (TC) were determined by an automatic biochemical analyzer (Chemray 240/800, Rayto Life and Analytical Sciences Co., Ltd., Shenzhen, China). Serum OLZ concentrations were determined by a high-performance liquid chromatography–electrospray ionization mass spectrometry (HPLC–MS/ESI) method we previously established in our laboratory (Zhou et al., 2004).

For the microbiota metabolites in the content of the rat cecum, the determination for 5-HT was performed according to the protocol provided by the rat 5-HT assay kit. Moreover, the concentrations of SCFAs were measured using a gas chromatography-mass spectrometry (GC-MS) equipped with an electron ionization (EI) source. In brief, an appropriate amount of cecal content sample was added to 2 ml of phosphoric acid-water (phosphoric acid: water = 1:3) solution and homogenized for 2 min. Two milliliters of ether were added to the sample for extraction for 10 min and centrifuged (4,000 r/min, 4°C), and the ether phase was removed and the aqueous phase was extracted again. The two extracts from ether phase were combined and evaporated to a constant volume of 2 ml, and then 2 μl of the sample was injected and analyzed. The analytes were separated with a capillary chromatographic column (HP-INNOWAX, 25 m × 0.20 mm × 0.40 μm). The sample was analyzed by GC-MS under the following conditions: the initial temperature was kept at 100°C for 5 min, linearly increased to 150°C at 5°C/min, and then fast increased to 240°C at 30°C/min, finally held at 240°C for 30 min. The mass spectrometer inlet temperature was 240°C, and the carrier gas flow rate was set at 1.0 ml/min. The EI source temperature was set to 200°C, and the collision energy was 70 eV.

The SCFAs content was calculated according to the formula:

w=(C ∗ V ∗ N)/(m ∗ M)

In the formula: *w*, the content of SCFAs in the rat cecum, in mmol/kg; C, the concentration of SCFAs in the test solution of the sample (measured value of the test solution by GC-MS), in mg/L; V, the constant volume, in the unit mL; N, dilution factor; m, weighed mass of the sample, in grams (g); M, molar mass of SCFAs, in g/mol.

### 2.4 16S rRNA high-throughput sequencing

DNA extraction from frozen fecal samples (180 mg) was subjected to use an PowerSoil^®^ DNA Isolation Kit (MOBIO, United States) according to the company’s protocols. After extracting the total DNA, specific primers with barcodes were synthesized according to the full-length primer sequences. After that, PCR amplification was performed and the products were purified, quantified and normalized to form a 16S rRNA sequencing library (SMRT Bell). After library quality inspection, qualified libraries were sequenced with PacBio Sequel. Data were analyzed by smart link analysis software. Then after exporting the PacBio off-board data as a CCS file, we first identified the CCS with a barcode, and obtained the Raw-CCS sequence data (lima v1.7.0). Using cut adapt 1.9.1, the raw sequence data were identified and removed of the primer sequence and filtered by length, to obtain a Clean-CCS sequences of primers. Finally, the chimera sequences were further identified and removed (UCHIME v4.2) to acquire an Effective-CCS sequences. The pretreated sequence files were quality-filtered by Trimmomatic (v 0.33) and FLASH (v 1.2.11). USEARCH (v10.0) was used to cluster the OTU, with 97% similarity truncation. The NCBI database was used for species annotation. The community richness (Chao1 richness estimator) and diversity (Shannon Index) were calculated using QIIME2. Beta diversity analysis was performed by the principal coordinate analysis (PCoA) method based on the R software package. The taxonomic groups (classes) and genera (subclasses) of bacteria represented by the differences between groups were identified by linear discriminant analysis (LDA) combined with an effector. The LEfSe criterion was set as LDA > 4 with *p* < 0.05. The *t*-test was performed on the species abundance data between groups using Metastats software.

### 2.5 Orexinogenic neuropeptides in the hypothalamus

#### 2.5.1 Western blot

Protein extraction was performed using RIPA buffer and PMSF, and protein quantification was performed using a BCA protein quantification kit. Proteins were separated using 10% SDS–PAGE and transferred to PVDF membranes (Millipore, Bedford, MA, United States). After blocking the membrane with 5% BSA for 1.5 h, the membrane was incubated with the corresponding primary antibodies (NPY and AgRP primary antibodies were diluted 1:1000 with antibody diluent, and β-actin was diluted 1:5000) for 12 h at 4°C. The membrane was then incubated with the secondary antibody for 90 min at room temperature. Using β-actin as an internal reference, the protein bands were quantified with ImageJ software.

#### 2.5.2 Quantitative real-time PCR

The samples were subjected to quantitative PCR using Applied Biosystems 7500/7500 Fast Real-Time PCR System and StepOnePlus Real-Time PCR System, and the primer sequences of the genes were as follows:

GAPDH, forward: 5′-ACA​GCA​ACA​GGG​TGG​TGG​AC-3′, reverse: 5′-TTT​GAG​GGT​GCA​GCG​AAC​TT-3′; NPY, forward: 5′-TAC​TCC​GCT​CTG​CGA​CAC​TA-3′, reverse: 5′-TGG​GGG​CAT​TTT​CTG​TGC​TT-3′; AgRP, forward: 5′-ACT​CTG​AAG​CTG​AAT​GCC​CAC-3′, reverse: 5′-CCC​ACA​CGT​GAC​TAC​TTC​CT-3′.

Total RNA was isolated from rat hypothalamus with TRIzol reagent according to the manufacturer’s instructions. Gene expression was calculated as △CT using GAPDH as a reference and was expressed relative to the control group normalized to a value of 1.

### 2.6 Statistical analysis

SPSS Statistics version 25.0 software (IBM Corp, Inc., Armonk, NY, United States) was used for data analysis. The results are expressed as the mean ± standard deviation (mean ± SD). The comparisons of repeated measures of WG% between saline and OLZ groups were made by multiple *t*-test with FDR determined using the two-stage linear step-up procedure of Benjamini, Krieger and Yekutieli. The abundance of SCFA metabolism-related microorganisms was compared in pairs of s-saline vs. s-olanzapine and v-saline vs. v-olanzapine, and serum levels of OLZ were compared between s-olanzapine and v-olanzapine groups, using Mann-Whitney U test. Before analyses, the Kolmogorov-Smirnov test was used to determine whether the relevant data are normally distributed. Then, Spearman’s test was considered as the way of testing data for heteroscedasticity. If the data of the four subgroups passed the normality test and had equal variances, two-way analysis of variance (ANOVA) followed by Tukey’s multiple comparisons test was utilized to compare the preselected pairs of groups (s-saline vs. s-olanzapine; v-saline vs. v-olanzapine; s-saline vs. v-saline; s-olanzapine vs. v-olanzapine). Otherwise, nonparametric Kruskal–Wallis ANOVA followed by Dunn’s multiple comparisons test was carried out. Graphs were made using GraphPad Prism 8 software.

## 3 Result

### 3.1 Olanzapine induces lipid disturbances in rats

The effects of olanzapine on body weight and metabolism of rats were analyzed by calculating WG%, WAT% and BAT% of rats. As shown in Figures 1C,D, in the sham-operated group, olanzapine administration from day 4 (t ratio = 2.304, *p* = 0.035) significantly increased the WG% relative to the saline group. However, under the condition of vagotomy, the olanzapine treatment had no significant effects on WG% compared with the saline group.

Under sham operation conditions, the olanzapine group had significantly increased rat WAT% (*p* = 0.030) and decreased BAT% (*p* = 0.005) when compared with the saline group (Figure 1E). Under the condition of vagus nerve resection, the WAT% and BAT% of the olanzapine group did not change significantly (Figure 1F). In addition, comparing the two olanzapine groups, the WAT% of the rats in the vagotomy group was significantly lower than that in the sham group (*p* = 0.001). This experiment also found that, regardless of whether the gut microbiota-brain axis was intact, olanzapine significantly increased serum TG levels in rats on average (s-saline vs. s-olanzapine, *p* = 0.015; v-saline vs. v-olanzapine, *p* = 0.002; Figure 1G). The integrity of the gut microbiota-brain axis did not affect the level of TG in rat serum. Meanwhile, olanzapine administration significantly increased the serum TC content of the vagotomy-treated rats (*p* = 0.022) but did not affect the serum TC content of the sham-operated rats (Figure 1H).

### 3.2 Olanzapine alters the gut microbial composition

**FIGURE 2:**
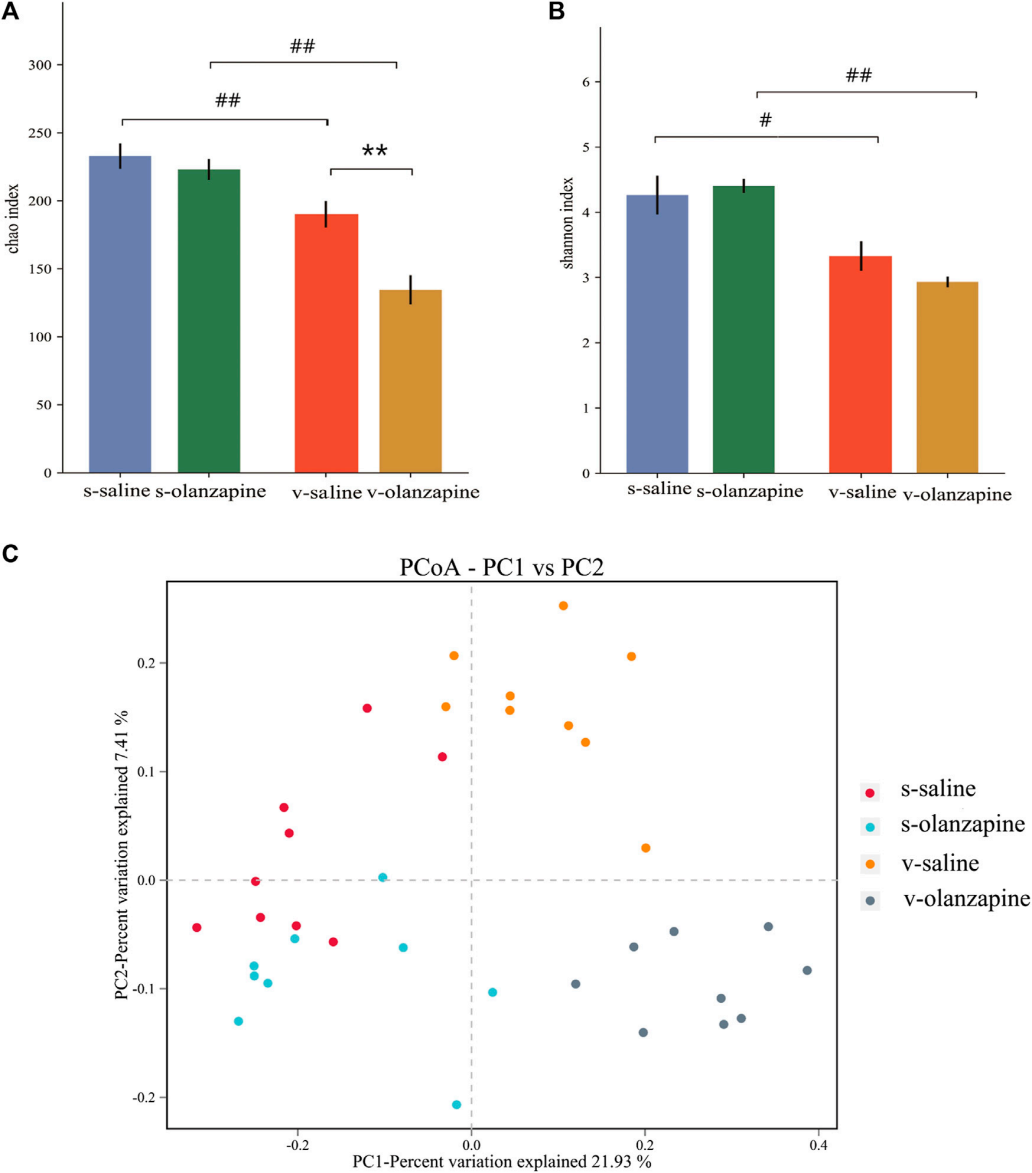
The results of species diversity analyses (OTU levels; **p < 0.01; ^#^ *p* < 0.05 and ^##^ *p* < 0.01) are shown among four groups as differences in the Chao1 index **(A)** and in the Shannon index **(B)**; and **(C)** PCoA analysis graph illustrating group profile clustering.

**FIGURE 3:**
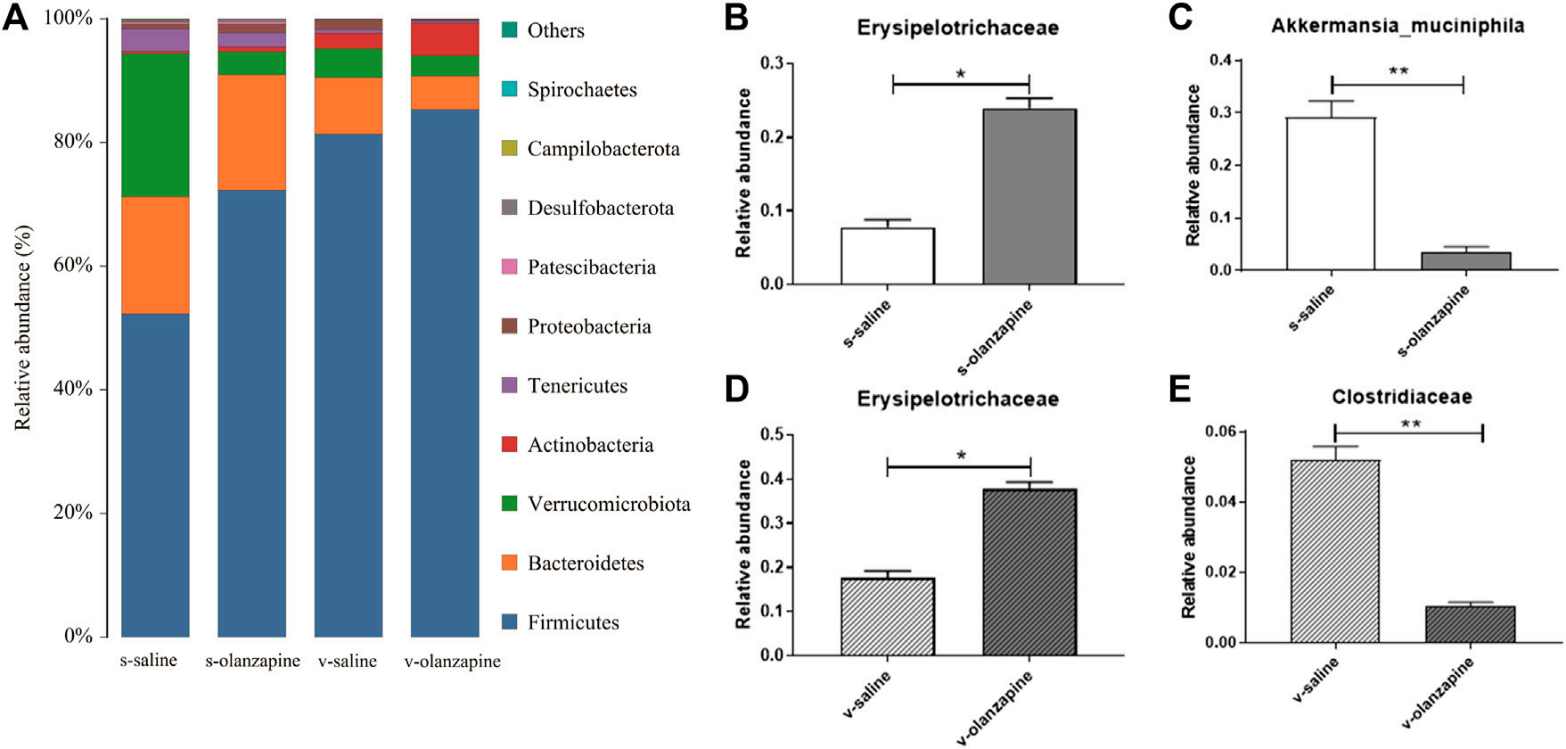
Gut microbial species distribution in the four groups: histograms showing species distribution at each phylum level **(A)**; difference analyses of Erysipelotrichaceae **(B)** and Akkermansia muciniphila **(C)** between the olanzapine-administered and saline groups under the sham-operated condition; and comparisons of Erysipelotrichaceae **(D)** and Clostridiaceae **(E)** between the olanzapine administrated and saline groups under the vagotomy condition. *p < 0.05 and **p < 0.01.

The effects of olanzapine and the vagus nerve on gut microbes were explored by analyzing the diversity and abundance of gut microbes in each group of rats. Under sham-operated conditions, olanzapine administration had no significant effect on the species abundance and β-diversity of gut microbes in rats. Under the condition of vagotomy, the olanzapine group significantly reduced the species abundance of rat gut microbes (*p* = 0.002, Figure 2A), and also showed a tendency to affect the β-diversity of rat gut microbes. In addition, the results indicated that gut microbial diversity and species abundance can be compromised by cutting off vagus nerve (Figures 2B,C), as revealed by comparisons of s-saline vs. v-saline (Chao1 index, *p* = 0.002; Shannon index, *p* = 0.003) and s-olanzapine vs. v-olanzapine (Chao1 index, *p* = 0.012; Shannon index, *p* = 0.002). Since the Firmicutes/Bacteroidetes ratio (F/B) was positively associated with obesity (Coras et al., 2019), we analyzed the ratio of F/B in each group (Figure 3A). As depicted, under the sham-operated condition the OLZ group had a significantly increased F/B ratio, whereas OLZ administration had little effect on F/B in the setting of vagotomy. In addition, with only saline administration, vagotomy also significantly increased the F/B ratio of the rats compared with the sham-operated group. We further analyzed the abundance of SCFA metabolism-related microorganisms in the four groups of gut microbiota (Figures 3B–E). Under sham-operated conditions, the OLZ group had a significantly increased abundance of Erysipelotrichaceae (*U* = 17, *p* = 0.040) and a significantly decreased abundance of *A. muciniphila* (*U* = 14, *p* = 0.019). After vagotomy, OLZ treatment significantly increased the abundance of Erysipelotrichaceae (*U* = 13, *p* = 0.014) and significantly decreased the abundance of Clostridiaceae in the gut microbiota (*U* = 7, *p* = 0.003).

**FIGURE 4:**
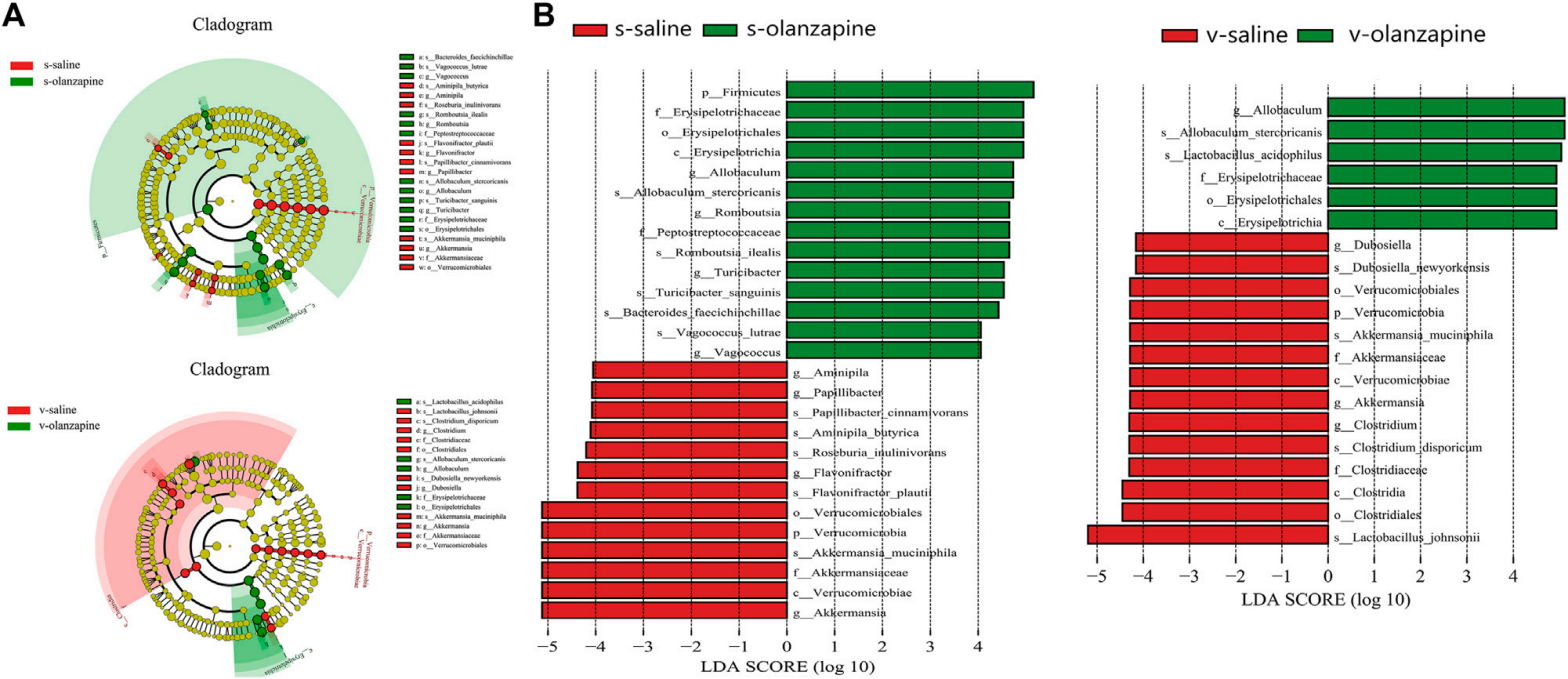
The LEfSe cladogram maps the distinct bacterial taxa between pairwise groups into a taxonomic tree **(A)**. The rings from inside to outside represent taxonomic levels from phylum to species. The diameter of each circle is proportional to the abundance of taxa. Colors of circles and shades indicate microbial lineages enriched in the corresponding samples, and p, c, o, f, g, and s stand for represent phylum, class, order, family, genus, and species, respectively. **(B)** Histograms of LEfSe LDA scores for different branches between groups. Only taxa with LDA values greater than 4 are shown. Colors (red or green) indicate that taxa are enriched in the corresponding taxa. The letters p, c, o, f, g, and s represent phylum, class, order, family, genus and species, respectively.

Figure 4A represents the LEfSe cladogram mapping the differential bacterial taxa between paired groups to taxonomic tree. Figure 4B shows the histogram of the LEfSe LDA scores computed for differential taxonomic clades between paired groups. The color (red or green) indicated the enrichment of the taxa within the corresponding groups. Under sham operation, the abundance of phylum Firmicutes was higher, and phylum Verrucomicrobiota was lower in the gut microbiota of OLZ treated group than saline group. However, when the rats were performed vagotomy, only the abundance of phylum Verrucomicrobiota was decreased in OLZ groups as compared with saline group.

### 3.3 Olanzapine increases the levels of short-chain fatty acids and 5-hydroxytryptamine in the cecum

**FIGURE 5:**
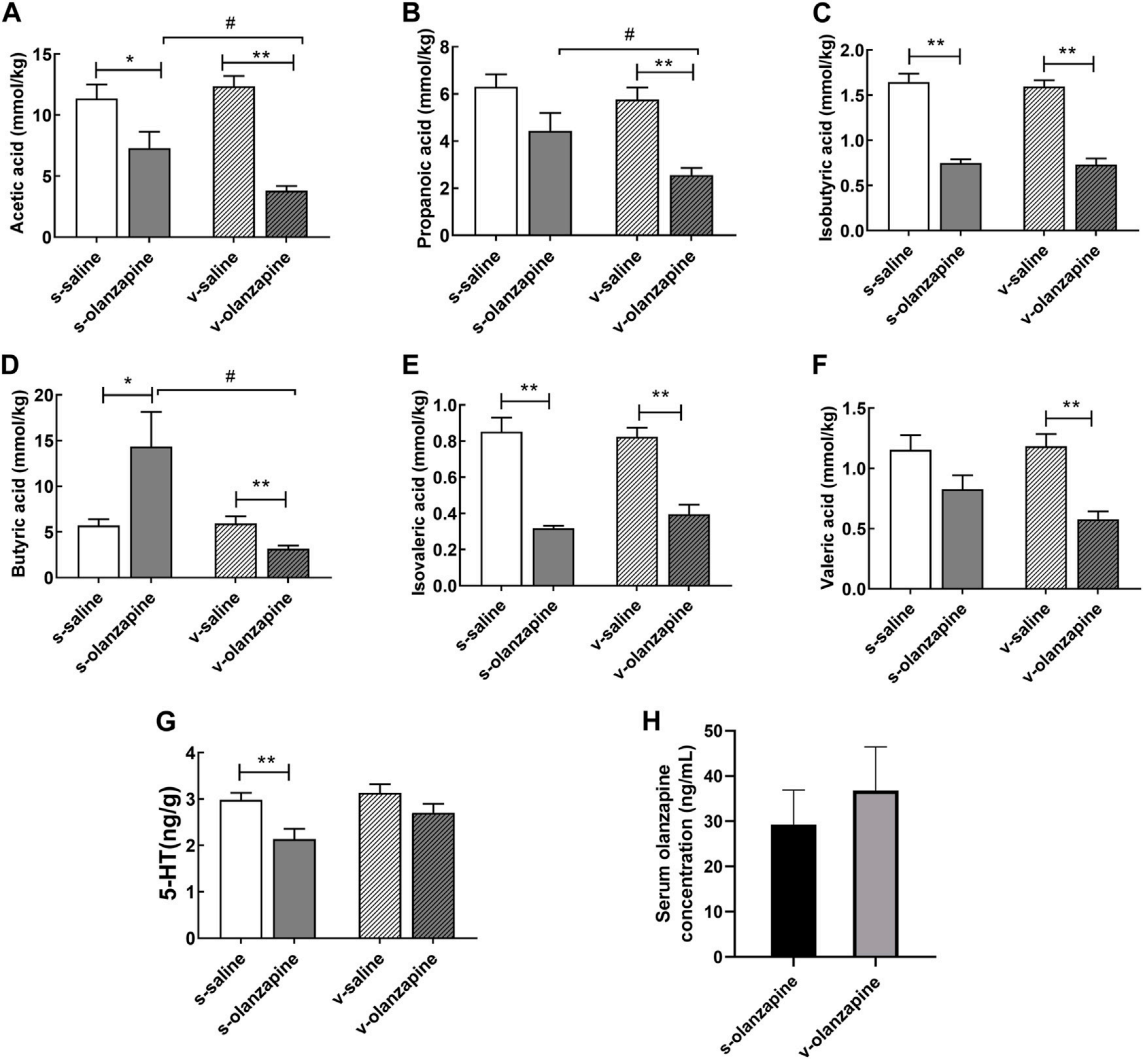
The concentrations of acetic acid **(A)**, propionic acid **(B)**, isobutyric acid **(C)**, butyric acid **(D)**, isovaleric acid **(E)**, valeric acid **(F)** and 5-HT **(G)** in the cecal contents among the four groups, and serum levels of olanzapine **(H)** between sham and vagotomy conditions, are depicted. *p < 0.05; **p < 0.01, ^#^ *p* < 0.05 and ^##^ *p* < 0.01.

To explore the effects of OLZ administration on SCFAs, the differences in the levels of six main SCFAs in the cecum are summarized in Figures 5A–F. In the case of sham operation, OLZ administration significantly reduced the levels of acetic acid (*p* = 0.036), isobutyric acid (*p* = 0.007), and isovaleric acid (*p* = 0.004) in the cecum and significantly increased the level of butyric acid (*p* = 0.033). In the vagus nerve resection group, olanzapine administration significantly reduced the content of six SCFAs in the cecum (acetic acid, *p* = 0.006; propanoic acid, *p* = 0.001; isobutyric acid, *p* = 0.005; butyric acid, *p* = 0.001; isovaleric acid, *p* = 0.009; valeric acid, *p* = 0.002). Under olanzapine administration, the vagus nerve resection group had significantly reduced levels of acetic acid (*p* = 0.019), propionic acid (*p* = 0.048) and butyric acid (*p* = 0.039) compared with the sham operation group. However, under the condition of just saline administration, the vagus nerve resection group and the sham operation group showed no significant differences in these SCFAs. As shown in Figure 5G, in the case of the sham operation, OLZ administration significantly reduced the 5-HT content in the cecum (*p* = 0.004). Under the circumstance of vagus nerve resection, the effects of OLZ on 5-HT in the cecum was not statistically significant. Since the pharmacokinetics of OLZ may vary significantly, we compared the serum levels of OLZ between s-olanzapine and v-olanzapine groups (Figure 5H) and no statistical difference was found (*U* = 16, *p* = 0.818).

### 3.4 Olanzapine regulates the expression of orexinogenic neuropeptides in the hypothalamus

**FIGURE 6:**
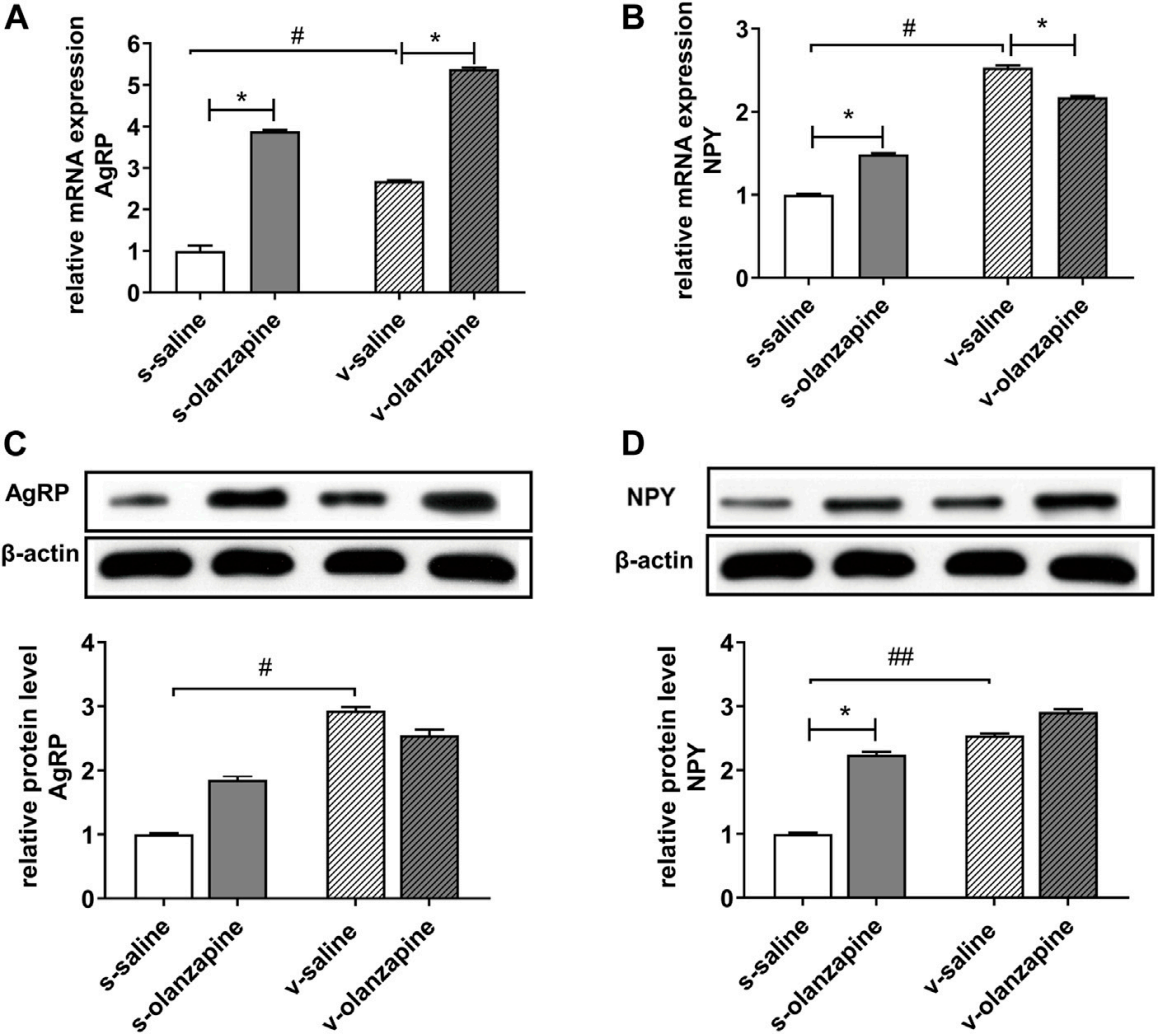
Different expressions in mRNA **(A,B)** and protein **(C,D)** levels of hypothalamic orexotropic-related neuropeptide Y/agouti-related peptide (NPY/AgRP). *p < 0.05, **p < 0.01, ^#^ *p* < 0.05 and ^##^ *p* < 0.01.

To further explore the specific mechanisms by which OLZ induces abnormal lipid metabolism and obesity through the gut microbiota-brain axis, we measured the mRNA and protein levels of orexins in the hypothalamus of different groups. As shown in Figure 6, the olanzapine group significantly increased the mRNA expression of the hypothalamic orexin neuropeptides NPY (*H* = 19.2, post-hoc *p* = 0.021) and AgRP (*H* = 30.9, post-hoc *p* = 0.042) and only significantly increased the protein level of NPY under sham-operated conditions (*H* = 24.6, post-hoc *p* = 0.016). In both vagotomy groups, the olanzapine group had decreased NPY mRNA expression (*H* = 19.2, post-hoc *p* = 0.049) but still upregulated AgRP mRNA expression (*H* = 30.9, post-hoc *p* = 0.033), but also had no significant effects on NPY and AgRP protein expression. Moreover, both the mRNA and protein levels of NPY and AgRP were significantly increased in the v-saline group compared with the s-saline group (NPY mRNA: *H* = 19.2, post-hoc *p* = 0.028; NPY protein: *H* = 24.6, post-hoc *p* = 0.006; AgRP mRNA: *H* = 30.9, post-hoc *p* = 0.015; AgRP protein: *H* = 27.2, post-hoc *p* = 0.034).

## 4 Discussion

In this study, the role of the gut microbiota-brain axis in olanzapine-induced lipid disturbances was systematically investigated for the first time in an animal model by severing the vagus nerve resection to disrupt gut-brain communication. We investigated the changes in lipid parameters induced by OLZ and inferred the effects of OLZ on gut microbiota by measuring the composition and abundance of gut microbes. We also discussed whether OLZ-induced alterations in SCFAs can activate the vagus nerve by increasing 5-HT, resulting in hypothalamic orexin expression.

The previous reports illustrate that even though OLZ promotes weight gain in humans, this effect seems to be gender-specific in rodent animal models, with OLZ-induced weight gain observed in female rats only, not male rats (Choi et al., 2007; Albaugh et al., 2011). Therefore, we chose female rats for our study. The results of this study found that the body weight of the rats in the v-olanzapine group had a tendency to increase, and olanzapine administration significantly increased the WG% of the sham-operated rats, which was consistent with the results in the literature (Kao et al., 2018). Olanzapine administration significantly increased WG% only when the vagus nerve was intact, suggesting that the intact gut microbiota-brain axis plays an important role in OLZ-induced weight gain in rats. Interestingly, the negative impact of the increase in WAT% and decrease in BAT% induced by OLZ administration was reversed by the disruption of the gut microbiota-brain axis (vagotomy), suggesting that OLZ-induced weight gain may require an intact gut microbiota-brain axis pathway. Previous studies have found that vagotomy reduces mitochondrial thermogenesis in brown adipose tissue (Andrews et al., 1985), indicating that the vagus nerve activation may have a certain inhibitory effect on brown adipose tissue mitochondrial function (Yang et al., 2007). Furthermore, OLZ significantly increased serum TG levels in rats regardless of whether the gut microbiota-brain axis was intact, consistent with another report (Chiu et al., 2006). The dyslipidemia caused by OLZ may due to a decrease in the metabolic rate or an increase in hepatic lipogenesis (Yang et al., 2007; Cai et al., 2015), which can be stratified to its peripheral mechanisms.

The abundance and diversity of gut microbes can be affected by OLZ, and the diversity of gut microbiota are more severely compromised when the vagus nerve was resected (Figures 2, 3). The results suggest that the vagus nerve allows the bidirectional communication between the gut and the brain, and has a key role in maintaining the homeostasis and diversity of gut microbes. In addition, we observed that OLZ increased the F/B of gut microbes by increasing the abundance of Firmicutes and decreasing the abundance of Bacteroidetes, which will become non-significant when gut microbiota-brain axis was disrupted by vagotomy. Brain exerts regulatory effects on gut microbes, as evident by the fact that mood and stress can affect the composition of gut microbes (Kim and Shin, 2018). Therefore, the changes in F/B may largely owing to the feedback top-down regulation of gut microbes by the brain during the treatment with OLZ. Disrupting the integrity of the vagus nerve in this experiment hindered the regulation of the gut microbiota by the brain, thereby causing no F/B changes after OLZ administration. In addition, it has been reported that F/B is positively correlated with obesity, and it has been found that when a prebiotic (B-COS^@^) is add-on to OLZ, it reduces the abundance of Firmicutes and alleviates OLZ-induced weight gain (Indiani et al., 2018; Kao et al., 2018). The above results suggest that the abnormal lipid metabolism and the increase in body fat caused by olanzapine are related to the increase in the F/B of gut microbes. In addition, OLZ administration significantly decreased the abundance of *A. muciniphila* in the gut of sham-operated rats. *A. muciniphila* is considered to be a promising probiotic and mainly promotes the generation of acetic, propionic, butyric, isobutyric, and isovaleric acids (Li et al., 2021). A decrease in its abundance is thought to be associated with metabolic disorders and inflammatory diseases, including obesity, type 2 diabetes, and inflammatory bowel disease (Zhang et al., 2019). Therefore, the results of this study suggest that OLZ-induced lipid disturbances are associated with a decrease in the abundance of *A. muciniphila* in the gut.

To further investigate whether OLZ affects 5-HT levels by altering the synthesis of SCFAs in the gut through the gut microbiota-brain axis, we determined the concentrations of SCFAs and 5-HT in the cecum content. Olanzapine can exert a negative impact on the gut microbiota to reduce the production of SCFAs, which in turn reduces 5-HT release and transmission from the vagus nerve to the brain. Therefore, the removal of the vagus nerve cuts off the bottom-up signal transmission of 5-HT related to the intestinal flora, but does not entirely affect the regulation of SCFAs by OLZ. Therefore, it is explainable that OLZ administration significantly reduced the levels of acetic acid, isobutyric acid, and isovaleric acid in the cecum of rats, no matter the rats were resected or not. However, among them, OLZ-induced reduction of acetic acid was aggravated when the vagus nerve was resected, reflecting certain protective role of top-down regulation from brain to gut microbiota. In support, OLZ-associated changes in propanoic acid and butyric acid were more severe in vagotomy group than in sham group. Interestingly, the effect of OLZ on butyrate in the gut seems to be closely related to the integrity of the vagus nerve. It has been shown that exogenous supplementation of butyrate can antagonize weight gain by reducing food intake and increasing energy metabolism (Li et al., 2018). Olanzapine administration significantly increased the concentration of butyric acid in the cecum of the s-olanzapine group, whereas decreased this SCFA of the v-olanzapine group. This result may be partially owing to the imbalanced modulation of OLZ on the abundance of butyrate-producing bacteria Erysipelotrichaceae and Clostridiaceae as indicated in Figure 3, because OLZ significantly increased the abundance of Erysipelotrichaceae in the gut of the s-olanzapine group. Although OLZ also significantly increased the abundance of Erysipelotrichaceae in the v-olanzapine group, the abundance of Clostridiaceae was simultaneously decreased in vagotomy group but not in the sham group, suggesting that the abundance of Clostridiaceae and related butyric acid yield may be specifically affected and more dependent on the integrity of the vagus nerve.

Changes in SCFA have been shown to affect 5-HT levels by affecting enterochromaffin cell activity. As shown in Figure 5, OLZ induced reductions in acetic acid and 5-HT in the sham-operated group, whereas only acetic acid reductions were observed in the vagotomy group. This suggests that olanzapine-induced reductions in acetic acid levels are mediated by the gut microbe-brain axis, further causing a decrease in 5-HT levels. 5-HT can stimulate vagal afferents through 5-HT_3_ receptors at vagal afferent terminals, and activation of vagal afferents can inhibit food intake in rats by increasing satiety (Laskiewicz et al., 2003; de Lartigue, 2016). Therefore, the results suggest that OLZ may ultimately affect the regulation of energy intake in the hypothalamus by affecting the gut microbiota-brain axis. By measuring the mRNA and protein levels of orexin NPY and AgRP in the hypothalamus, we confirmed that OLZ further affects the levels of NPY and AgRP through the vagus nerve and may further aggravate the increase in body fat content in rats by affecting energy intake. It is worth noting that when the vagus nerve was resected, the OLZ-related changes of NPY and AgRP in protein levels were disappeared. The results suggest that impairment in the vagus nerve itself may also lead to dysregulation of energy metabolism, thereby aggravating the influence of external factors (e.g., AAPD) on lipid metabolism and energy intake in the body. Nevertheless, as regard to OLZ-induced lipid disturbances, there may be some differences between real schizophrenia patients and normal rats we used. Notably, both impaired vagal afferent and efferent signaling has been implicated in the pathophysiology of schizophrenia (Klarer et al., 2018). Because schizophrenia patients may experience disrupted function of vagus nerve and cannot properly regulate downstream gut microbiota, this factor may contribute to the predisposition of abnormal gut microbiota and metabolic disturbances in schizophrenia patients before AAPD treatment. Recently, the changes in gut microbiota in patients with schizophrenia taking OLZ have been studied (Pelka-Wysiecka et al., 2019). Using an animal model of vagotomy, herein we further explored how the impact of OLZ on the abundance and diversity of gut microbes can contribute to its lipidemia side effects through gut microbiota-brain axis.

There are several limitations of this study. First, the effects of OLZ on TG and TC seem to be independent of the gut microbiota-brain axis during the course of the study. Considering that TG and TC levels may be more related to hepatic lipid metabolism, the peripheral role of gut microbiota during AAPD treatment needs to be further investigated. Second, this study did not involve the treatment on gut microbiota, in future studies combinations of probiotics/prebiotics with OLZ will be considered to explore the detailed mechanisms of and novel intervention strategy to AAPD related metabolic side effects. Third, the pharmacokinetics of OLZ can vary significantly in rats and may account for the differences between the two OLZ-treated groups. However, in our study, no significant difference in the serum concentrations of OLZ was found. The pharmacokinetics of OLZ cannot fully explain the differences in the indicators of weight gain, WAT%, the community richness and diversity of gut microbiota, and certain SCFAs levels (acetic acid, propanoic acid and butyric acid) between s-olanzapine and v-olanzapine groups.

**FIGURE 7:**
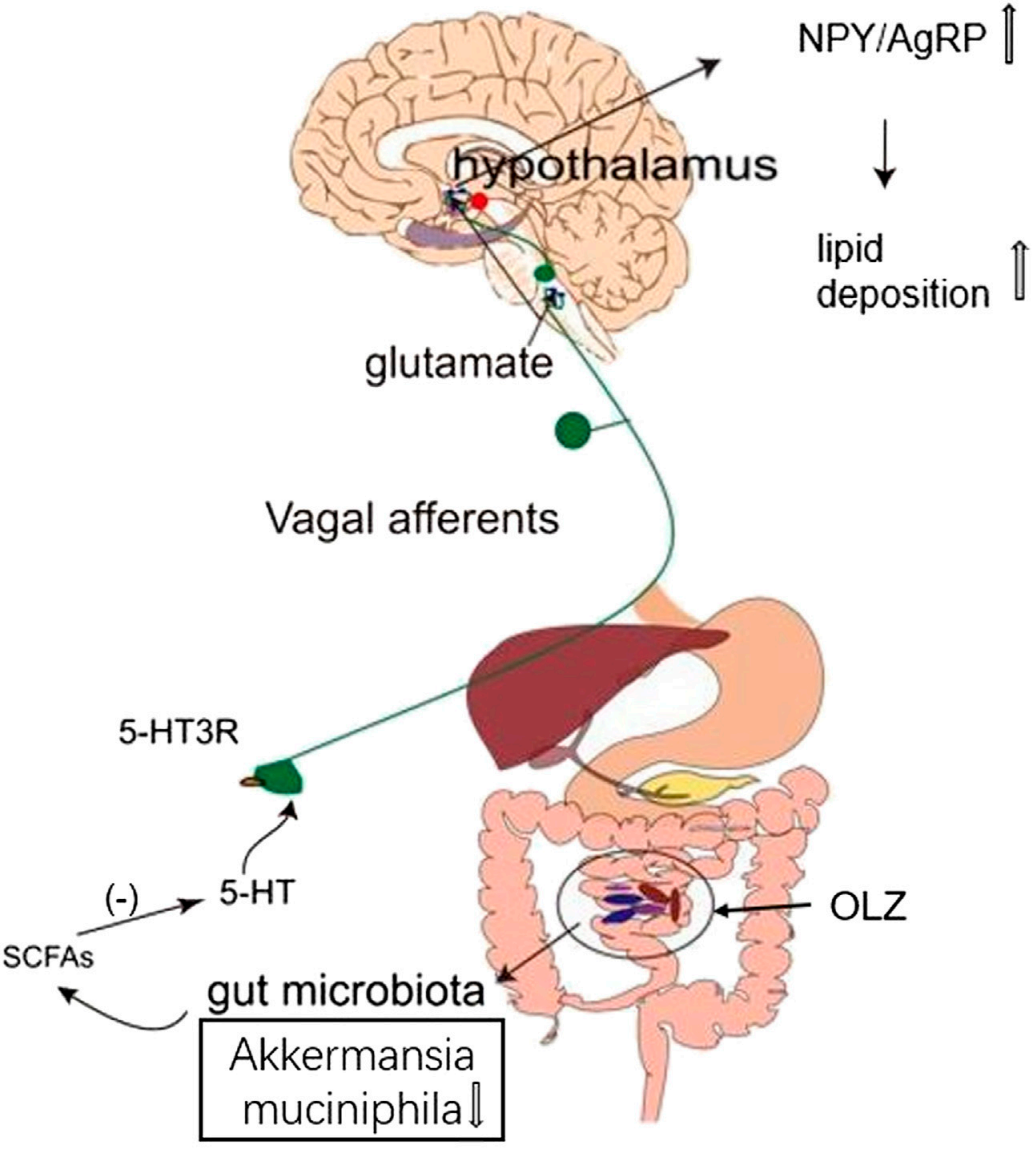
This study suggests a possible scenario that olanzapine can induce changes in the levels of short-chain fatty acids (SCFAs) by altering the abundance and composition of gut microbiota, thereby reducing 5-HT secretion in the gut and related glutamatergic signal transduction through vagus nerve, which increases the ratio of hypothalamic orexotropic-related neuropeptide Y/agouti-related peptide (NPY/AgRP), eventually in turn contributing to accumulated lipid deposition in rats.

In conclusion, OLZ administration is at least partially responsible for obesity by increasing F/B ratio, and this effect requires an intact gut microbiota-brain axis. In addition, OLZ may alter the levels of the microbial metabolite SCFAs by reducing the abundance of *A. muciniphila* in the gut. Its alterations lead to lower levels of 5-HT, which stimulate the activity of orexotropic neurons in the hypothalamus *via* the gut microbiota-brain axis to induce lipid disturbances (Figure 7). A better understanding of the peripheral and central mechanisms underlying OLZ-induced lipid disturbances could shed light on forming new strategy to ameliorate the metabolic side effects of antipsychotic treatment.

## Data Availability

The datasets presented in this study can be found in online repositories. The names of the repository/repositories and accession number(s) can be found below: https://www.ncbi.nlm.nih.gov/; PRJNA816822.

## References

[B1] Abbas M. M. Soto P. Ramalingam L. El-Manzalawy Y. Bensmail H. Moustaid-Moussa N. (2022). Sex differences in fish oil and olanzapine effects on gut microbiota in diet-induced obese mice. Nutrients 14 (2), 349. 10.3390/nu14020349 35057526 PMC8780445

[B2] Ader M. Kim S. P. Catalano K. J. Ionut V. Hucking K. Richey J. M. (2005). Metabolic dysregulation with atypical antipsychotics occurs in the absenceof underlying disease: A placebo-controlled study of olanzapine and risperi-done in dogs. Diabetes 54, 862–871. 10.2337/diabetes.54.3.862 15734866

[B3] Albaugh V. L. Judson J. G. She P. Lang C. H. Maresca K. P. Joyal J. L. (2011). Olanzapine promotes fat accumulation in male rats by decreasing physical activity, repartitioning energy and increasing adipose tissue lipogenesis while impairing lipolysis. Mol. Psychiatry 16 (5), 569–581. 10.1038/mp.2010.33 20308992 PMC2892549

[B4] Andrews P. L. Rothwell N. J. Stock M. J. (1985). Effects of subdiaphragmatic vagotomy on energy balance and thermogenesis in the rat. J. Physiol. 362, 1–12. 10.1113/jphysiol.1985.sp015658 3894621 PMC1192877

[B5] Anyanwagu U. Mamza J. Donnelly R. Idris I. (2018). Effects of obesity on metabolic and cardiovascular outcomes following insulin initiation in patients with type 2 diabetes. Obes. Res. Clin. Pract. 12 (1), 72–84. 10.1016/j.orcp.2017.08.005 28939465

[B6] Ballon J. S. Pajvani U. B. Mayer L. E. Freyberg Z. Freyberg R. Contreras I. (2018). Pathophysiology of drug induced weight and metabolic effects: Findings from an RCT in healthy volunteers treated with olanzapine, iloperidone, or placebo. J. Psychopharmacol. 32 (5), 533–540. 10.1177/0269881118754708 29444618 PMC6996198

[B7] Bilgic S. Tastemir Korkmaz D. Azirak S. Guvenc A. N. Kocaman N. Ozer M. K. (2017). The protective effect of thymoquinone over olanzapine-induced side effects in liver, and metabolic side effects. Bratisl. Lek. Listy 118 (10), 618–625. 10.4149/BLL_2017_119 29198130

[B8] Browning K. N. (2015). Role of central vagal 5-HT3 receptors in gastrointestinal physiology and pathophysiology. Front. Neurosci. 9, 413. 10.3389/fnins.2015.00413 26578870 PMC4625078

[B9] Bush N. D. Townsend L. K. Wright D. C. (2018). AICAR prevents acute olanzapine-induced disturbances in glucose homeostasis. J. Pharmacol. Exp. Ther. 365 (3), 526–535. 10.1124/jpet.118.248393 29581153

[B10] Cai H. L. Tan Q. Y. Jiang P. Dang R. L. Xue Y. Tang M. M. (2015). A potential mechanism underlying atypical antipsychotics-induced lipid disturbances. Transl. Psychiatry 5 (10), e661. 10.1038/tp.2015.161 26485545 PMC4930135

[B11] Canfora E. E. Jocken J. W. Blaak E. E. (2015). Short-chain fatty acids in control of body weight and insulin sensitivity. Nat. Rev. Endocrinol. 11 (10), 577–591. 10.1038/nrendo.2015.128 26260141

[B12] Casey D. E. Zorn S. H. (2015). The pharmacology of weight gain with antipsychotics. J. Clin. Psychiatry 62 (7), 4–10. 11346195

[B13] Chiu C. C. Chen K. P. Liu H. C. Lu M. L. (2006). The early effect of olanzapine and risperidone on insulin secretion in atypical-naive schizophrenic patients. J. Clin. Psychopharmacol. 26 (5), 504–507. 10.1097/01.jcp.0000237947.80764.d9 16974193

[B14] Choi S. DiSilvio B. Unangst J. Fernstrom J. D. (2007). Effect of chronic infusion of olanzapine and clozapine on food intake and body weight gain in male and female rats. Life Sci. 81 (12), 1024–1030. 10.1016/j.lfs.2007.08.009 17822717 PMC3998666

[B15] Coccurello R. Moles A. (2010). Potential mechanisms of atypical antipsychotic-induced metabolic derangement: Clues for understanding obesity and novel drug design. Pharmacol. Ther. 127 (3), 210–251. 10.1016/j.pharmthera.2010.04.008 20493213

[B16] Coras R. Kavanaugh A. Boyd T. Huynh D. Lagerborg K. A. Xu Y.-J. (2019). Choline metabolite, trimethylamine N-oxide (TMAO), is associated with inflammation in psoriatic arthritis. Clin. Exp. Rheumatol. 37, 481–484. 30620278 PMC6529247

[B17] Cork S. C. (2018). The role of the vagus nerve in appetite control: Implications for the pathogenesis of obesity. J. Neuroendocrinol. 30 (11), e12643. 10.1111/jne.12643 30203877

[B18] Crane J. D. Palanivel R. Mottillo E. P. Bujak A. L. Wang H. Ford R. J. (2015). Inhibiting peripheral serotonin synthesis reduces obesity and metabolic dysfunction by promoting Brown adipose tissue thermogenesis. Nat. Med. 21 (2), 166–172. 10.1038/nm.3766 25485911 PMC5647161

[B19] Cryan J. F. O'Riordan K. J. Cowan C. S. M. Sandhu K. V. Bastiaanssen T. F. S. Boehme M. (2019). The microbiota-gut-brain Axis. Physiol. Rev. 99 (4), 1877–2013. 10.1152/physrev.00018.2018 31460832

[B20] Davey K. J. Cotter P. D. O'Sullivan O. Crispie F. Dinan T. G. Cryan J. F. (2013). Antipsychotics and the gut microbiome: Olanzapine-induced metabolic dysfunction is attenuated by antibiotic administration in the rat. Transl. Psychiatry 3, e309. 10.1038/tp.2013.83 24084940 PMC3818006

[B21] de Lartigue G. (2016). Role of the vagus nerve in the development and treatment of diet-induced obesity. J. Physiol. 594 (20), 5791–5815. 10.1113/JP271538 26959077 PMC5063945

[B22] Duca F. A. Lam T. K. (2014). Gut microbiota, nutrient sensing and energy balance. Diabetes Obes. Metab. 16 (1), 68–76. 10.1111/dom.12340 25200299

[B23] Eckburg P. B. Bik E. M. Bernstein C. N. Purdom E. Dethlefsen L. Sargent M. (2005). Diversity of the human intestinal microbial flora. Science 308 (5728), 1635–1638. 10.1126/science.1110591 15831718 PMC1395357

[B24] Grajales D. Ferreira V. Valverde A. M. (2019). Second-generation antipsychotics and dysregulation of glucose metabolism: Beyond weight gain. Cells 8 (11), E1336. 10.3390/cells8111336 31671770 PMC6912706

[B25] Halford J. C. Harrold J. A. Lawton C. L. Blundell J. E. (2005). Serotonin (5-HT) drugs: Effects on appetite expression and use for the treatment of obesity. Curr. Drug Targets 6 (2), 201–213. 10.2174/1389450053174550 15777190

[B26] Huang D. Gao J. Li C. Nong C. Huang W. Zheng X. (2021). A potential probiotic bacterium for antipsychotic-induced metabolic syndrome: Mechanisms underpinning how Akkermansia muciniphila subtype improves olanzapine-induced glucose homeostasis in mice. Psychopharmacol. Berl. 238 (9), 2543–2553. 10.1007/s00213-021-05878-9 34046717

[B27] Huang M. Panos J. J. Kwon S. Oyamada Y. Rajagopal L. Meltzer H. Y. (2014). Comparative effect of lurasidone and blonanserin on cortical glutamate, dopamine, and acetylcholine efflux: Role of relative serotonin (5-HT)2A and DA D2 antagonism and 5-HT1A partial agonism. J. Neurochem. 128 (6), 938–949. 10.1111/jnc.12512 24164459

[B28] Ikegami M. Ikeda H. Ohashi T. Kai M. Osada M. Kamei A. (2013). Olanzapine-induced hyperglycemia: Possible involvement of histaminergic, dopaminergic and adrenergic functions in the central nervous system. Neuroendocrinology 98 (3), 224–232. 10.1159/000356119 24135197

[B29] Indiani C. Rizzardi K. F. Castelo P. M. Ferraz L. F. C. Darrieux M. Parisotto T. M. (2018). Childhood obesity and firmicutes/bacteroidetes ratio in the gut microbiota: A systematic review. Child. Obes. 14 (8), 501–509. 10.1089/chi.2018.0040 30183336

[B30] Isaacson R. H. Beier J. I. Khoo N. K. Freeman B. A. Freyberg Z. Arteel G. E. (2020). Olanzapine-induced liver injury in mice: Aggravation by high-fat diet and protection with sulforaphane. J. Nutr. Biochem. 81, 108399. 10.1016/j.jnutbio.2020.108399 32388251 PMC7263947

[B31] Kao A. C. Chan K. W. Anthony D. C. Lennox B. R. Burnet P. W. (2019). Prebiotic reduction of brain histone deacetylase (HDAC) activity and olanzapine-mediated weight gain in rats, are acetate independent. Neuropharmacology 150, 184–191. 10.1016/j.neuropharm.2019.02.014 30763656

[B32] Kao A. C. Spitzer S. Anthony D. C. Lennox B. Burnet P. W. J. (2018). Prebiotic attenuation of olanzapine-induced weight gain in rats: Analysis of central and peripheral biomarkers and gut microbiota. Transl. Psychiatry 8 (1), 66. 10.1038/s41398-018-0116-8 29540664 PMC5852210

[B33] Kim Y. K. Shin C. (2018). The microbiota-gut-brain Axis in neuropsychiatric disorders: Pathophysiological mechanisms and novel treatments. Curr. Neuropharmacol. 16 (5), 559–573. 10.2174/1570159X15666170915141036 28925886 PMC5997867

[B34] Klarer M. Arnold M. Gunther L. Winter C. Langhans W. Meyer U. (2014). Gut vagal afferents differentially modulate innate anxiety and learned fear. J. Neurosci. 34 (21), 7067–7076. 10.1523/JNEUROSCI.0252-14.2014 24849343 PMC6608191

[B35] Klarer M. Krieger J. P. Richetto J. Weber-Stadlbauer U. Gunther L. Winter C. (2018). Abdominal vagal afferents modulate the brain transcriptome and behaviors relevant to schizophrenia. J. Neurosci. 38 (7), 1634–1647. 10.1523/JNEUROSCI.0813-17.2017 29326171 PMC6705869

[B36] Laskiewicz J. Królczyk G. Zurowski G. Sobocki J. Matyja A. Thor P. J. (2003). Effects of vagal neuromodulation and vagotomy on control of food intake and body weight in rats. J. Physiol. Pharmacol. 54 (4), 603–610. 14726614

[B37] Li Y. Zhao X. Feng X. Liu X. Deng C. Hu C. H. (2016). Berberine alleviates olanzapine-induced adipogenesis via the AMPKα-SREBP pathway in 3T3-L1 cells. Int. J. Mol. Sci. 17 (11), 1865. 10.3390/ijms17111865 PMC5133865 27834848

[B38] Li Z. Yi C. X. Katiraei S. Kooijman S. Zhou E. Chung C. K. (2018). Butyrate reduces appetite and activates Brown adipose tissue via the gut-brain neural circuit. Gut 67 (7), 1269–1279. 10.1136/gutjnl-2017-314050 29101261

[B39] Li Z. Hu G. Zhu L. Sun Z. Jiang Y. Gao M. J. (2021). Study of growth, metabolism, and morphology of Akkermansia muciniphila with an *in vitro* advanced bionic intestinal reactor. BMC Microbiol. 21 (1), 61. 10.1186/s12866-021-02111-7 33622254 PMC7901181

[B40] Liu X. M. Zhao X. M. Deng C. Zeng Y. P. Hu C. H. (2019). Simvastatin improves olanzapine-induced dyslipidemia in rats through inhibiting hepatic mTOR signaling pathway. Acta Pharmacol. Sin. 40 (8), 1049–1057. 10.1038/s41401-019-0212-1 30728467 PMC6786380

[B41] Lord C. C. Wyler S. C. Wan R. Castorena C. M. Ahmed N. Mathew D. (2017). The atypical antipsychotic olanzapine causes weight gain by targeting serotonin receptor 2C. J. Clin. Invest. 127 (9), 3402–3406. 10.1172/JCI93362 28805659 PMC5669575

[B42] Marder S. R. Cannon T. D. (2019). Schizophrenia. N. Engl. J. Med. 381 (18), 1753–1761. 10.1056/NEJMra1808803 31665579

[B43] Maruvada P. Leone V. Kaplan L. M. Chang E. B. (2017). The human microbiome and obesity: Moving beyond associations. Cell Host Microbe 22 (5), 589–599. 10.1016/j.chom.2017.10.005 29120742

[B44] Mayer E. A. Tillisch K. Gupta A. (2015). Gut/brain axis and the microbiota. J. Clin. Invest. 125 (3), 926–938. 10.1172/jci76304 25689247 PMC4362231

[B45] Morgan A. P. Crowley J. J. Nonneman R. J. Quackenbush C. R. Miller C. N. Ryan A. K. (2014). The antipsychotic olanzapine interacts with the gut microbiome to cause weight gain in mouse. PLoS One 9 (12), e115225. 10.1371/journal.pone.0115225 25506936 PMC4266663

[B46] Oh C. M. Namkung J. Go Y. Shong K. E. Kim K. Kim H. (2015). Regulation of systemic energy homeostasis by serotonin in adipose tissues. Nat. Commun. 6, 6794. 10.1038/ncomms7794 25864946 PMC4403443

[B47] Pelka-Wysiecka J. Kaczmarczyk M. Baba-Kubis A. Liskiewicz P. Wronski M. Skonieczna-Zydecka K. (2019). Analysis of gut microbiota and their metabolic potential in patients with schizophrenia treated with olanzapine: Results from a six-week observational prospective cohort study. J. Clin. Med. 8 (10), E1605. 10.3390/jcm8101605 31623359 PMC6832832

[B48] Reigstad C. S. Salmonson C. E. Rainey J. F. 3rd Szurszewski J. H. Linden D. R. Sonnenburg J. L. (2015). Gut microbes promote colonic serotonin production through an effect of short-chain fatty acids on enterochromaffin cells. FASEB J. 29 (4), 1395–1403. 10.1096/fj.14-259598 25550456 PMC4396604

[B49] Rummel-Kluge C. Schuster T. Peters S. Kissling W. (2008). Partial compliance with antipsychotic medication is common in patients with schizophrenia. Aust. N. Z. J. Psychiatry 42 (5), 382–388. 10.1080/00048670801961107 18473256

[B50] Savontaus E. Conwell I. M. Wardlaw S. L. (2002). Effects of adrenalectomy on AGRP, POMC, NPY and CART gene expression in the basal hypothalamus of fed and fasted rats. Brain Res. 958 (1), 130–138. 10.1016/s0006-8993(02)03674-0 12468037

[B51] Schwartz M. W. Woods S. C. Porte D. Seeley R. J. Baskin D. G. (2000). Central nervous system control of food intake. Nature 404 (6778), 661–671. 10.1038/35007534 10766253

[B52] Singh A. de la Serre C. de Lartigue G. (2020). Gut microbiota sPARk vagus nerve excitation. J. Physiol. 598 (11), 2043–2044. 10.1113/JP279763 32187377 PMC7266700

[B53] Skonieczna-Zydecka K. Loniewski I. Misera A. Stachowska E. Maciejewska D. Marlicz W. (2019). Second-generation antipsychotics and metabolism alterations: A systematic review of the role of the gut microbiome. Psychopharmacol. Berl. 236 (5), 1491–1512. 10.1007/s00213-018-5102-6 PMC6598971 30460516

[B54] Tecott L. H. (2007). Serotonin and the orchestration of energy balance. Cell Metab. 6 (5), 352–361. 10.1016/j.cmet.2007.09.012 17983581

[B55] Teff K. L. Rickels M. R. Grudziak J. Fuller C. Nguyen H. L. Rickels K. (2013). Antipsychotic-induced insulin resistance and postprandial hormonal dysregulation independent of weight gain or psychiatric disease. Diabetes 62 (9), 3232–3240. 10.2337/db13-0430 23835329 PMC3749337

[B56] Teff K. L. Kim S. F. (2011). Atypical antipsychotics and the neural regulation of food intake and peripheral metabolism. Physiol. Behav. 104 (4), 590–598. 10.1016/j.physbeh.2011.05.033 21664918 PMC3139777

[B57] Visconti A. Le Roy C. I. Rosa F. Rossi N. Martin T. C. Mohney R. P. (2019). Interplay between the human gut microbiome and host metabolism. Nat. Commun. 10 (1), 4505. 10.1038/s41467-019-12476-z 31582752 PMC6776654

[B58] Walther D. J. Peter J. U. Winter S. Höltje M. Paulmann N. Grohmann M. (2003). Serotonylation of small GTPases is a signal transduction pathway that triggers platelet alpha-granule release. Cell 115 (7), 851–862. 10.1016/s0092-8674(03)01014-6 14697203

[B59] Yabut J. M. Crane J. D. Green A. E. Keating D. J. Khan W. I. Steinberg G. R. (2019). Emerging roles for serotonin in regulating metabolism: New implications for an ancient molecule. Endocr. Rev. 40 (4), 1092–1107. 10.1210/er.2018-00283 30901029 PMC6624793

[B60] Yamashita H. Maruta H. Jozuka M. Kimura R. Iwabuchi H. Yamato M. (2009). Effects of acetate on lipid metabolism in muscles and adipose tissues of type 2 diabetic Otsuka Long-Evans Tokushima Fatty (OLETF) rats. Biosci. Biotechnol. Biochem. 73 (3), 570–576. 10.1271/bbb.80634 19270372

[B61] Yang L. H. Chen T. M. Yu S. T. Chen Y. H. (2007). Olanzapine induces SREBP-1-related adipogenesis in 3T3-L1 cells. Pharmacol. Res. 56 (3), 202–208. 10.1016/j.phrs.2007.05.007 17651982

[B62] Zhang T. Li Q. Cheng L. Buch H. Zhang F. (2019). Akkermansia muciniphila is a promising probiotic. Microb. Biotechnol. 12 (6), 1109–1125. 10.1111/1751-7915.13410 31006995 PMC6801136

[B63] Zhou Z. Li X. Li K. Xie Z. Cheng Z. Peng W. (2004). Simultaneous determination of clozapine, olanzapine, risperidone and quetiapine in plasma by high-performance liquid chromatography-electrospray ionization mass spectrometry. J. Chromatogr. B Anal. Technol. Biomed. Life Sci. 802 (2), 257–262. 10.1016/j.jchromb.2003.11.037 15018785

[B64] Zmora N. Suez J. Elinav E. (2019). You are what you eat: Diet, health and the gut microbiota. Nat. Rev. Gastroenterol. Hepatol. 16 (1), 35–56. 10.1038/s41575-018-0061-2 30262901

